# Revisiting the Taxonomic Status of the Biomedically and Industrially Important Genus *Amycolatopsis*, Using a Phylogenomic Approach

**DOI:** 10.3389/fmicb.2018.02281

**Published:** 2018-09-27

**Authors:** Vartul Sangal, Michael Goodfellow, Jochen Blom, Geok Yuan Annie Tan, Hans-Peter Klenk, Iain C. Sutcliffe

**Affiliations:** ^1^Faculty of Health and Life Sciences, Northumbria University, Newcastle upon Tyne, United Kingdom; ^2^School of Natural and Environmental Sciences, Newcastle University, Newcastle upon Tyne, United Kingdom; ^3^Bioinformatics and Systems Biology, Justus-Liebig-Universität, Gießen, Germany; ^4^Institute of Biological Sciences, Faculty of Science, University of Malaya, Kuala Lumpur, Malaysia

**Keywords:** prokaryotic systematics, core genome, average nucleotide identity (ANI), average amino-acid identity (AAI), digital DNA-DNA hybridization, *Amycolatopsis*

## Abstract

Strains belonging to the genus *Amycolatopsis* are well known for the production of a number of important antimicrobials and other bioactive molecules. In this study, we have sequenced the genomes of five *Amycolatopsis* strains including *Amycolatopsis circi* DSM 45561^T^, *Amycolatopsis palatopharyngis* DSM 44832^T^ and *Amycolatopsis thermalba* NRRL B-24845^T^. The genome sequences were analyzed with 52 other publically available *Amycolatopsis* genomes, representing 34 species, and 12 representatives from related genera including *Saccharomonospora*, *Saccharopolyspora*, *Saccharothrix*, *Pseudonocardia* and *Thermobispora*. Based on the core genome phylogeny, *Amycolatopsis* strains were subdivided into four major clades and several singletons. The genus *Amycolatopsis* is homogeneous with only three strains noted to group with other genera. *Amycolatopsis halophila* YIM93223^T^ is quite distinct from other *Amycolatopsis* strains, both phylogenetically and taxonomically, and belongs to a distinct genus. In addition, *Amycolatopsis palatopharyngis* DSM 44832^T^ and *Amycolatopsis marina* CGMCC4 3568^T^ grouped in a clade with *Saccharomonospora* strains and showed similar taxogenomic differences to this genus as well as other *Amycolatopsis* strains. The study found a number of strains, particularly those identified as *Amycolatopsis orientalis*, whose incorrect identification could be resolved by taxogenomic analyses. Similarly, some unclassified strains could be assigned with species designations. The genome sequences of some strains that were independently sequenced by different laboratories were almost identical (99–100% average nucleotide and amino acid identities) consistent with them being the same strain, and confirming the reproducibility and robustness of genomic data. These analyses further demonstrate that whole genome sequencing can reliably resolve intra- and, inter-generic structures and should be incorporated into prokaryotic systematics.

## Introduction

The genus *Amycolatopsis* is well known for the commercial production of multiple antibiotics, including the important broad spectrum antibiotics rifamycin and, vancomycin (Xu et al., 2014; Chen et al., 2016). These strains also have the potential to produce a number of other secondary metabolites and bioactive molecules (Adamek et al., 2018) and, can be exploited for a range of biotechnological applications (Davila Costa and Amoroso, 2014). The genus currently includes 72 validly named species (List of prokaryotic names with standing in nomenclature^1^) that may vary in their phenotypic and morphological characteristics (Tan and Goodfellow, 2015). *Amycolatopsis* strains commonly reside in arid, or hyper-arid soil and have chemo-organotrophic to facultatively autotrophic lifestyles (Tan and Goodfellow, 2015). However, some species have also been isolated from activated sludge, equine placentas and from clinical and plant material (Tan and Goodfellow, 2015). *Amycolatopsis* strains can be mesophilic or thermophilic, with a DNA GC content of 66–75 mol%. They can form branching substrate hyphae that fragment into square, or rod-shaped elements and carry aerial hyphae (Saintpierre-Bonaccio et al., 2005; Tan and Goodfellow, 2015).

A recent multilocus sequence analysis (MLSA) based on seven housekeeping genes (*atpD*, *clpB*, *gapA*, *gyrB*, *nuoD*, *pyrH* and *rpoB*) revealed the presence of four major groups of species within the genus, with some singletons (Adamek et al., 2018), whilst Sanchez-Hidalgo et al. (2018) described 11 subgroups in a 16 S rRNA analysis, and four major groupings based on an MLSA with four housekeeping genes (*atpD, dnaK, recA* and *rpoB*). The type strains of *Amycolatopsis halophila* and *Amycolatopsis marina* were found to be quite distinct from other *Amycolatopsis* strains. Although average nucleotide identities based on MUMmer (ANIm) supported the classification of these strains within the genus, the percentage of conserved proteins indicated that *A. halophila* might belong to a different genus (Adamek et al., 2018). A five gene MLSA (with *atpI*, *gyrA*, *ftsZ*, *secA* and *dnaK*) also indicated that *A. halophila* is more similar to members of the genus *Prauserella* than to *Amycolatopsis* species (Bose et al., 2016). Therefore, we have applied a more comprehensive phylogenetic and taxogenomic approach to the genus (Sangal et al., 2016) and analyzed 57 *Amycolatopsis* genome sequences belonging to 34 species, including five sequenced by us in this study. Twelve genome sequences representing other genera, including *Saccharomonospora*, *Saccharopolyspora*, *Saccharothrix*, *Pseudonocardia*, and *Thermobispora*, were included for the comparative analyses (**Supplementary Table 1**). This study highlights the subgeneric structure within the genus and supports the separation of *A. halophila* as a member of a different genus. In addition, it is clear that a number of strains have been misidentified and misclassified using traditional approaches. Some strains sequenced independently by different laboratories grouped together, suggesting that genome based approaches are more reliable and reproducible than techniques such as DNA–DNA hybridisation, which suffers from a lack of reproducibility and compatibility of results between different laboratories (Achtman and Wagner, 2008; Moore et al., 2010).

## Materials and Methods

### Bacterial Strains and Genome Sequencing

Five strains, *Amycolatopsis circi* DSM 45561^T^, *Amycolatopsis palatopharyngis* DSM 44832^T^, *Amycolatopsis ruanii* 49.3a, *Amycolatopsis thermalba* 50.9 b and *A. thermalba* NRRL B-24845^T^ were grown in 5 ml Brain-Heart Infusion broth (Oxoid) at 28°C for 48 h. Genomic DNA from each strain was extracted from 1.5 ml culture using the UltraClean^®^ Microbial DNA Isolation Kit (MoBio).

The genome sequencing was performed on an Illumina MiSeq instrument and the paired-end reads were assembled using SPAdes 3.9.0 (Bankevich et al., 2012). The draft genome sequences have been submitted to the DDBJ/EMBL/GenBank databases and are publicly available (**Supplementary Table 1**).

The genome sequences of 52 *Amycolatopsis* strains and 12 representative strains of related genera were obtained from GenBank (**Supplementary Table 1**). Two independent genome assemblies were available for seven *Amycolatopsis* strains in GenBank and both of them were included to test the reliability and reproducibility of the genomic data.

### Computational Analyses

BLAST-based pairwise average nucleotide identity (ANIb) and pairwise fragment similarity scores (fragment size of 500-bp) were calculated from the nucleotide sequences using Jspecies (Richter and Rosselló-Móra, 2009) and GEGENEES (Agren et al., 2012), respectively. The genome sequences were annotated using Prokka (Seemann, 2014) and were compared using EDGAR (Blom et al., 2016) for calculation of the core- and pan- genomes and the number of genes shared within each phylogenetic cluster. Pairwise amino acid identity (AAI) was also calculated using EDGAR (Blom et al., 2016) and pairwise digital DNA–DNA hybridization values were calculated using GGDC 2.1 (Auch et al., 2010a,b). A maximum-likelihood (ML) tree was constructed from the concatenated protein sequences of the core genes after removing sites with missing data using the best-fit amino acid substitution model (LG + F + I + G4) with 100,000 SH-aLRT and 100,000 ultrafast bootstrap replicates using IQ-Tree (Nguyen et al., 2015). A Neighbor-Joining (NJ) tree was generated from the pairwise GGDC distance matrix using MEGA (Kumar et al., 2016). The tree was re-rooted on *Thermobispora bispora* DSM 43833^T^. Pairwise percentage of conserved proteins (POCP) were calculated using the scripts, data_file_4.sh (Moose, 2017) and runPOCP.sh (Pantiukh and Grouzdev, 2017) that are based on the previously described approach (Qin et al., 2014).

## Results

### Phylogenetic and Taxogenomic Groups Within the Genus

**FIGURE 1:**
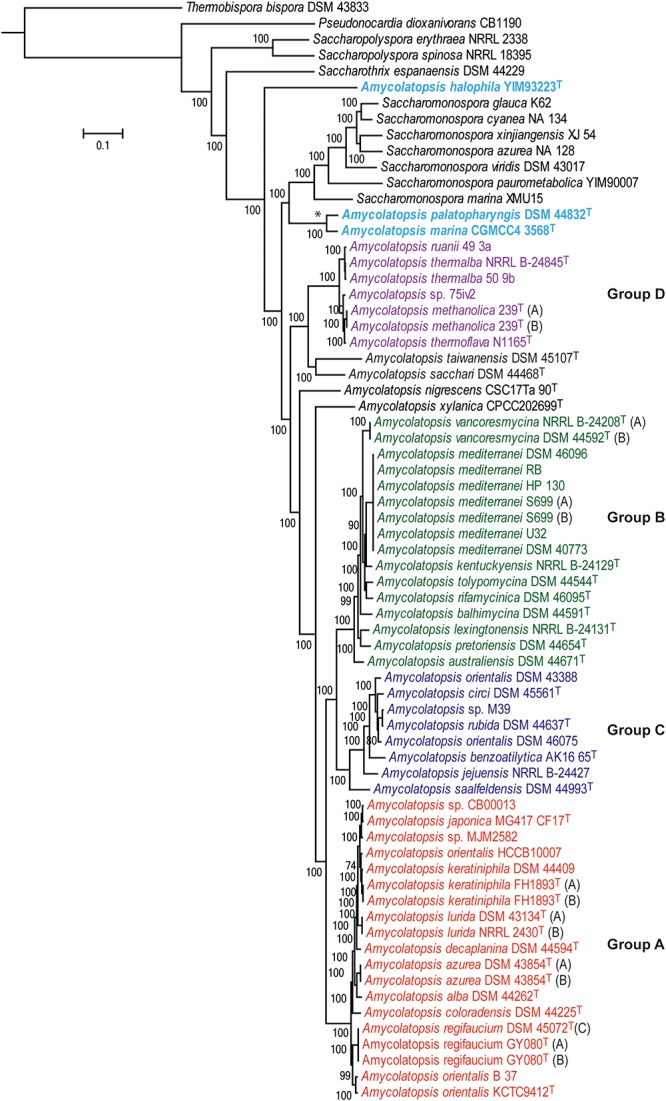
A maximum-likelihood tree derived from concatenated core protein sequences. The scale bar represents amino acid substitutions per site. *Amycolatopsis* groups are labeled and shown in different colors. The clade with the two *Amycolatopsis* strains that are more close to *Saccharomonospora* strains is labeled with a star (^∗^) sign.

A total of 602 genes were conserved (core genome) across the 69 genomes, including the strains representing related genera (**Supplementary Table 1**). A ML tree generated from concatenated core proteins resolved *Amycolatopsis* strains, representing 34 species (including 29 type strains), into four major groups and several singletons (**Figure 1**). These groupings are consistent with both the MLSA based phylogenies albeit with minor exceptions (Adamek et al., 2018; Sanchez-Hidalgo et al., 2018). We have applied the same group designations as used by Adamek et al. (2018). Group A is the largest group with 19 isolates assigned to nine species, including two without formal species designations. Group B encompasses 16 isolates (nine species) while groups C and D are relatively smaller with eight isolates (6 species and 1 undefined) and seven isolates (four species and one undefined), respectively (**Figure 1**). *Amycolatopsis taiwanensis* DSM 45107^T^, *Amycolatopsis sacchari* DSM 44468^T^, *Amycolatopsis nigrescens* CSC17Ta 90^T^ and *Amycolatopsis xylanica* CPCC202699^T^ are present as singletons within the *Amycolatopsis* clade (**Figure 1**). These strains were also recovered as singletons in the MLSA analysis of Sanchez-Hidalgo et al. (2018), except for *A. xylanica* which was located at the periphery of the strains in our cluster A. Of the other singletons, *A. sacchari* DSM 44468^T^ is consistently associated with *A. dongchuanensis* in 16S rRNA gene trees [e.g. (Wang et al., 2018); group I in Sanchez-Hidalgo et al. (2018) and Tang et al. (2016)], whilst the *A. nigrescens* is probably closely related to *Amycolatopsis minnesotensis* (Tang et al., 2016; Sanchez-Hidalgo et al., 2018;Wang et al., 2018). Similarly, *A. taiwanensis* is consistently associated with *Amycolatopsis helveola* and *Amycolatopsis pigmentata* (Tang et al., 2016; Sanchez-Hidalgo et al., 2018; Wang et al., 2018). These associations suggest that these singletons may expand into species groups as more whole genomes become available.

Notably, *A. palatopharyngis* DSM 44832^T^ and *A. marina* CGMCC4 3568^T^ [16S and MLSA group G (Sanchez-Hidalgo et al., 2018)] formed a group that is more closely related to *Saccharomonospora* strains than to other members of *Amycolatopsis* while *A. halophila* YIM93223^T^ [16S group J (Sanchez-Hidalgo et al., 2018)] is quite distant to all other strains and forms a single member clade (**Figure 1**). Overall, group structure is also consistent with the NJ tree obtained from the BLAST-based genome-to-genome distances (**Supplementary Figure 1**).

**FIGURE 2:**
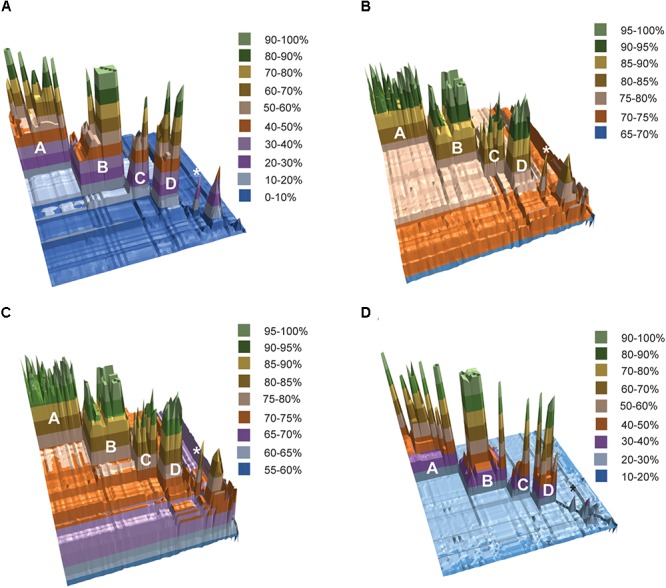
3D plots of pairwise matrices obtained from **(A)** fragmented BLAST searches (FBS values), **(B)** average nucleotide identities (ANIb values), **(C)** average amino-acid identities (AAI values) and **(D)** digital DNA-DNA hybridisation (dDDH values). The *Amycolatopsis* groups are labeled. The group of *A. palatopharyngis* DSM 44832^T^ and *A. marina* CGMCC4 3568^T^ is labeled with the star (^∗^) sign.

The results of fragmented genome BLAST searches are consistent with the phylogenetic groupings with a mean fragmented BLAST similarity (FBS) within the *Amycolatopsis* groups varying from 35.9 ± 18.8 for Group C to 65.1 ± 17.0 for Group D (**Figure 2A**). The mean FBS score between the *Amycolatopsis* groups varied from 8.6 ± 1.8 to 11.4 ± 2.1 and between *Amycolatopsis* (excluding the three anomalous strains *A. palatopharyngis* DSM 44832^T^, *A. marina* CGMCC4 3568^T^, and *A. halophila* YIM93223^T^) and strains of the other genera from 3.9 ± 1.6. Although the group of *A. palatopharyngis*, and *A. marina* is closely related to *Saccharomonospora* strains, the mean FBS score (5.3 ± 1.2) between these taxa was comparable to the score with the other *Amycolatopsis* strains (5.6 ± 1.1). Similarly, *A. halophila* was also equidistant from the *Amycolatopsis* strains (4.1 ± 1.1) and from the other genera (including *A. marina* and *A. palatopharyngis*; 3.5 ± 1.0).

Average nucleotide identity and AAI values also support the phylogenetic groupings (**Figures 2B,C**). Average ANIb values within and between the *Amycolatopsis* groups are >80% (85.94 ± 5.24 - 93.65 ± 3.41) and >75% (75.68 ± 0.69 - 76.97 ± 0.91), respectively. The strains of the other genera and the three anomalous strains shared lower ANIb values when compared with the *Amycolatopsis* strains (72.52 ± 1.52 and 72.77 ± 1.77, respectively). Similarly, average AAI values are >80% (86.69 ± 6.11 - 94.48 ± 3.06) within the *Amycolatopsis* groups and approximately 70%, or higher (70.99 ± 1.02 - 73.93 ± 1.98) between these groups. Average AAI values between *Amycolatopsis* strains and the three anomalous strains and the other genera are 67.54 ± 4.27 and 66.45 ± 3.70, respectively.

Pairwise dDDH values were also calculated for the dataset which varied between 36.2 ± 14.4 and 59.5 ± 19.7 within each *Amycolatopsis* group (**Figure 2D**). The average dDDH values ranged from 21.4 ± 0.5 to 22.2 ± 0.5 between the *Amycolatopsis* groups which is comparable to the values between the *Amycolatopsis* strains and the strains from the other genera (20.1 ± 0.7). This confirms that dDDH is useful in identifying strains belonging to the same species but has limited resolution at the intergeneric level.

A POCP value of 50% is often used as the genus boundary where two strains with more than 50% conserved proteins are considered to belong to the same genus (Qin et al., 2014). Consistent with this, pairwise POCP values among the *Amycolatopsis* strains (excluding anomalous strains) varied between 50.71 and 99.96% (67.38 ± 9.75; **Supplementary Table 2**). However, POCP could not resolve the status of the anomalous strains as *A. marina* and *A. palatopharyngis* type strains showed POCP values of 57.09 ± 2.95 and 58.53 ± 3.13 against the strains in the *Amycolatopsis* clade and the *Saccharomonospora* strains, respectively. POCP values between *A. halophila* YIM93223^T^, and other *Amycolatopsis* strain was <50% except for some of the strains in group D, *A. marina* and *A. palatopharyngis* (**Supplementary Table 2**). All of these results suggest that 54 of the 57 *Amycolatopsis* strains belong to the same genus while *A. halophila* YIM93223^T^ should be assigned to a different genus. *A. palatopharyngis* DSM 44832^T^ and *A. marina* CGMCC4 3568^T^ grouped in the clade with *Saccharomonospora* strains; however, they showed comparable distances to both *Saccharomonospora* and *Amycolatopsis* strains. A larger analysis including more species from the genus *Saccharomonospora* and other representatives of the family *Pseudonocardiaceae* is required to clarify the status of these two species. However, it is notable that the recent phylogenomic study of Nouioui et al. (2018) recovered *A. marina* within the genus *Amycolatopsis*, although this study did not include *A. palatopharyngis*.

### Genomic Features of *Amycolatopsis* Strains Are Consistent With the Phylogenetic Groups

**FIGURE 3:**
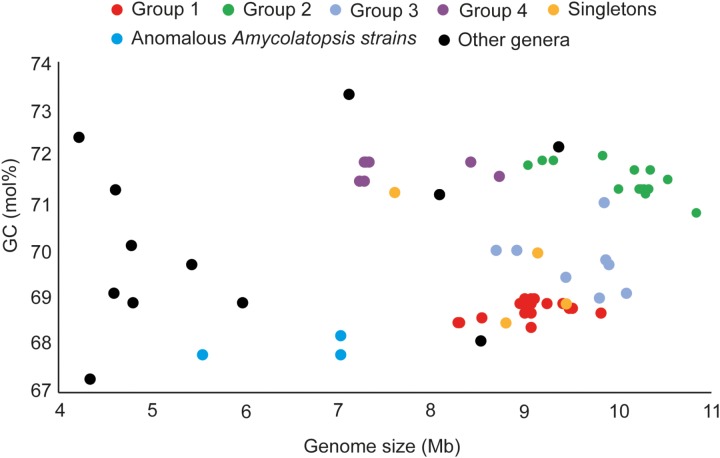
Distribution of genome sizes and GC contents among the different phylogenetic groups.

The genome sizes of the *Amycolatopsis* strains varied between 7 and 11 Mb with a GC content of 68 – 72 mol% (**Figure 3** and **Supplementary Table 1**). *A. halophila* YIM93223^T^ is clearly an outlier with a genome size of 5.6 Mb and a 67.8 mol% GC content. *Amycolatopsis salitolerans* consistently clusters with *A. halophila* in 16S rRNA gene trees (Guan et al., 2012; Tang et al., 2016; Sanchez-Hidalgo et al., 2018; Wang et al., 2018) and thus likely belongs to the same genus. *A. salitolerans* is reported to have a GC content of 66.4% (Guan et al., 2012); whilst this value needs confirmation from whole genome sequence data, it is similar to that for *A. halophila* YIM93223^T^ and is outwith the range for strains of *Amycolatopsis sensu stricto* (**Figure 3**). Cumulatively, these data are consistent with the recent proposal that *A. halophila* and *A. salitolerans* should be reclassified into the genus *Haloechinothrix* (Tang et al., 2010) as *Haloechinothrix halophila* comb. nov. and *Haloechinothrix salitolerans* comb. nov., respectively (Nouioui et al., 2018). *A. palatopharyngis* DSM 44832^T^ and *A. marina* CGMCC4 3568^T^ are also separable due to genomes sizes and GC content being at the lower end of the respective range for the *Amycolatopsis* strains (**Figure 3** and **Supplementary Table 1**). Although the range of the genome size and GC content is quite broad for *Amycolatopsis sensu stricto* strains, some patterns are visible at the group level. For instance, the strains in Group A have a genome size of 8.27 – 9.81 Mb, and a GC content of 68.5 – 69 mol%. The genomes of strains in Group B varied between 9.04 and 10.86 Mb with a GC content ranging from 70.8 to 72 mol%.

**FIGURE 4:**
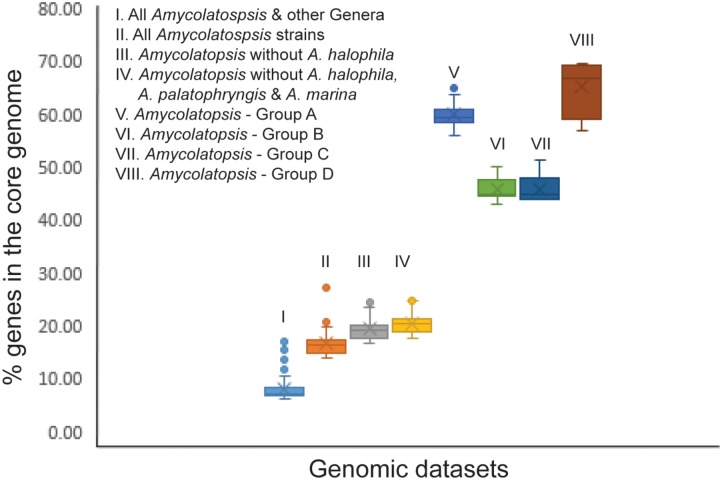
A box plot showing the fraction of genes in the core genome for the different genomic datasets.

In order to establish whether the size of the core genome can help identify intergeneric boundaries, core genes were calculated for the entire dataset followed by a sequential removal of the other genera and the outlier strains *A. halophila*, *A. palatopharyngis* and *A. marina*. The core genome of the entire dataset contained 602 genes (6.1–16.9%). The number of annotated genes among the *Amycolatopsis* strains ranged between 5,098 and 9,890 after excluding those of other genera (**Supplementary Table 1**) with 1,382 of them (14.0 – 27.1%) shared by all of the strains, which compares well with the 1,212 core genes identified by Adamek et al. (2018). The number of core genes increased to 1,634 (17.5 – 24.7% of 6,606 – 9,890 genes) after removing the *A. halophila* strain from the dataset and only slightly to 1,739 genes (17.6 – 25%) after exclusion of the genomes of the *A. palatopharyngis* and *A. marina* strains. Therefore, the proportion of genes in the core genome may not be reliable for separating members of different genera due to overlap in values within a genus and when strain(s) from other genera are included (**Figure 4**). As expected, the number of core genes is much higher within each *Amycolatopsis* group (43.1 – 69.5%).

### Species-Group A

Group A is the largest of the *Amycolatopsis* groups including nine type strains (**Figure 1** and **Supplementary Table 1**), many of which are known to produce different antibiotics and other bioactive molecules (Tan and Goodfellow, 2015). Average FBS, ANIb, and AAI values within this group are 55.9 ± 12.4, 90.76 ± 2.88, and 92.48 ± 2.56, respectively. dDDH values are consistent with the recognition of nine species, namely *Amycolatopsis alba*, *Amycolatopsis azurea*, *Amycolatopsis coloradensis*, *Amycolatopsis decaplanina*, *Amycolatopsis japonica*, *Amycolatopsis keratiniphila*, *Amycolatopsis lurida*, *Amycolatopsis orientalis*, and *Amycolatopsis regifaucium* (**Figure 1**). This analysis identified a potential case of misidentification of the industrial vancomycin producer *A. orientalis* strain HCCB10007 (Xu et al., 2014), which, as noted previously (Adamek et al., 2018; Sanchez-Hidalgo et al., 2018), is notably distant from *A. orientalis* KCTC9412^T^ (dDDH 38.4%; ANIb 50.8%) and shows a dDDH of 72% and ANIb of 96.4% with *A. keratiniphila* FH1893^T^. Therefore, this strain should be reclassified as *A. keratiniphila*. Two strains, *Amycolatopsis* sp. CB00013 and *Amycolatopsis* sp. MJM252 could be assigned to *A. japonica* based on the dDDH cut-off value of 70%, as noted previously (Sanchez-Hidalgo et al., 2018). These assignments are also consistent with the previously suggested FBS score of >66.8% and ANIb and AAI values of ∼95% or higher (Konstantinidis and Tiedje, 2005b; Sangal et al., 2016). While *A. keratiniphila*, and *A. japonica* strains are clearly separated into two species by a dDDH value of ∼60%, other matrices suggest that they may belong to the same species.

Multiple assemblies were available in GenBank for four of the type strains in Group A (**Figure 1** and **Supplementary Table 1**) and all of them were included to check the reproducibility of the sequence data between different laboratories and the robustness of the approach. Pairwise FBS, ANIb, AAI, and dDDH values between these assembles were >99.7%, confirming the authenticity of these strains albeit with a minor exception. The dDDH value between the genome sequences of *A. keratiniphila* FH1893^T^ (Assembly accession numbers CA_900105855.1, and GCA_001953855.1) was 97.7%. These assemblies were submitted by two different laboratories and this variation may reflect the quality of the sequences. Other taxogenomic values are consistent with them being associated with the same strain (**Supplementary Tables 3A–D**).

### Species-Group B

Group B is also quite diverse encompassing 16 of the 57 *Amycolatopsis* strains (**Figure 1** and **Supplementary Table 1**). The mean FBS (55.74 ± 21.36), ANIb (90.45 ± 4.72) and, AAI scores (91.23 ± 4.40) for this group are comparable to the values observed for Group A. All of the pairwise matrices support the presence of nine species including *Amycolatopsis australiensis*, *Amycolatopsis balhimycina*, *Amycolatopsis kentuckyensis*, *Amycolatopsis lexingtonensis*, *Amycolatopsis mediterranei*, *Amycolatopsis pretoriensis*, *Amycolatopsis rifamycinica*, *Amycolatopsis tolypomycina*, and *Amycolatopsis vancoresmycina* in the group, which is consistent with their taxonomic assignment (**Figure 1** and **Supplementary Table 1**). While most of the members of this clade are known to produce antibiotics and/or bioactive molecules (Davila Costa and Amoroso, 2014; Tan and Goodfellow, 2015), the *A. kentuckyensis*, *A. lexingtonensis*, and *A. pretoriensis* strains were isolated from lesions on equine placenta and may be pathogenic (Labeda et al., 2003). *A. vancoresmycina* DSM 44592^T^ has been sequenced independently by two laboratories and the high genomic similarities are consistent with them being the same strain (**Supplementary Tables 3A–D**). This group also includes seven *A. mediterranei* isolates that are grouped together with >97.7% dDDH, and 99.8–100% ANIb and AAI similarities, indicating it to be a highly homogeneous species with very limited genomic diversity.

### Species-Group C

Group C shows a slightly higher diversity than was found in Groups A and B (FBS, 35.85 ± 18.83; ANIb, 85.94 ± 5.24; AAI, 86.69 ± 6.11; **Supplementary Tables 3A–D**). Based on the taxogenomic matrices, eight strains within this group can be assigned to seven species (**Figure 1** and **Supplementary Table 1**). The assignment of five of these species, *A. circi*, *Amycolatopsis rubida*, *Amycolatopsis benzoatilytica*, *Amycolatopsis jejuensis* and *Amycolatopsis saalfeldensis* is consistent with the literature (Tan and Goodfellow, 2015). However, two strains, DSM 43388 and DSM 46075, identified as *A. orientalis*, are clearly misclassified. The type strain of *A. orientalis*, KCTC9412^T^, clustered in Group A. Both these strains are quite distinct from each other as well as from other strains in Group C and belong to novel species based on the taxogenomic values. *Amycolatopsis* sp. strain M39 shares a dDDH value of 93.2, FBS value of 87.25 ± 0.17, ANIb 98.96 ± 0.10, and AAI of 99.1 with *A. rubida* DSM 44637^T^ suggesting that strain M39 strain should be assigned to this species, as noted previously (Sanchez-Hidalgo et al., 2018). Most of the strains in this group were isolated from diverse soil samples but with some exceptions, e.g., *A. benzoatilytica* strain AK16 65^T^, was isolated from a patient with submandibular mycetoma (Tan and Goodfellow, 2015).

### Species-Group D

The species group D is the smallest group with an average FBS score of 65.12 ± 16.95, an average ANIb value of 93.65 ± 3.41 and an average AAI score of 94.48 ± 3.06 (**Supplementary Tables 3A–D**). These strains were assigned to four species based on the pairwise dDDH values including *Amycolatopsis methanolica*, *A. thermalba*, *Amycolatopsis thermoflava* and a potentially novel species with *Amycolatopsis* sp. strain 75iv2 (**Supplementary Table 3D**). *A. ruanii* strain 49.3a probably should be reclassified as *A. thermalba* due to its high dDDH 93.3%, FBS 92.78 ± 0.12, ANIb 99.19 ± 0.04, and AAI value of 99.23 with the type strain of latter. Both genome sequences of the *A. methanolica* type strain show ≥99.9% ANIb and AAI similarities but their dDDH is slightly lower (98.3%). Similar to *A. keratiniphila*, this may be due to minor variations in the quality of genome sequences generated in different laboratories. Although, *Amycolatopsis* sp. strain 75iv2 and *A. thermoflava* strain N1165^T^ can be assigned to two different species based on the dDDH values, they share ANIb (95.08%) and AAI (96.42%) with each other that marginally overlap using the recommended cut-off for defining species (Konstantinidis and Tiedje, 2005a, 2007; Sangal et al., 2016).

## Discussion

Whole genome based approaches are now routinely used to resolve the structure of complex prokaryotic taxa (Kyrpides et al., 2014; Sangal et al., 2014, 2016; Schleifer et al., 2015; Sutcliffe, 2015; Mahato et al., 2017; Carro et al., 2018; Chun et al., 2018). In addition to the genome-based phylogenies, calculation of dDDH, ANI and AAI from the genomic sequences have become the gold standard for defining species with cut-off values of 70%, 95% and 95–96%, respectively (Konstantinidis and Tiedje, 2005a, 2007; Auch et al., 2010b). However, the data on separating prokaryotic genera are limited (Qin et al., 2014; Sangal et al., 2016). We have previously suggested that an ANI value of approximately 74.8% can be used to define genera and that FBS values of ∼66.8% and 6.9% can help identifying interspecific and intergeneric boundaries, respectively (Sangal et al., 2016). Using a combination of phylogenomic and taxogenomic approaches, we defined seven species groups in the genus *Rhodococcus* that were as distant from each other as from representatives of other genera (Sangal et al., 2016). In contrast, strains representing the genus *Micromonospora* were found to be monophyletic, consistent with their assignment to a single genus (Carro et al., 2018). In this study, we have extended this approach to the industrially and biomedically important genus, *Amycolatopsis*, and found that the majority of strains assigned to this taxon clustered on a single branch that was separated from the related genus *Saccharomonospora* (**Figure 1**). However, the type strains of *A. palatopharyngis* and *A. marina* clustered more closely to *Saccharomonospora* and the type strain of *A. halophila* formed a single member clade (**Figure 1**). The taxogenomic matrices are in agreement with the phylogenomic groupings (**Figure 2**) with minor exceptions. For example, the FBS and ANI values between *A. taiwanensis* strain DSM 45107^T^ and the other *Amycolatopsis* strains are slightly below the suggested cut-off values (**Supplementary Tables 3A,B**); however, this strains clustered close to group D within the *Amycolatopsis* clade and clearly belongs to this genus. Therefore, the suggested cut-off values should be considered a guide and used in combination with genome based phylogenies (Sangal et al., 2016).

The percentage of conserved proteins (POCP) has been used to define strains at the genus level with ≥50% proteins between a pair of strains with at least 50% alignable region and 40% sequence identity considered to indicate membership of the same genus (Qin et al., 2014). However, the status of the anomalous strains *A. marina* and *A. palatopharyngis* remains unresolved due to POCP values of > 50% both with the other *Amycolatopsis* strains as well as with the *Saccharomonospora* strains (**Supplementary Table S2**). As noted above, further analysis of a larger dataset is required not only to clarify the taxonomic status of *A. marina* and *A. palatopharyngis* but also that of the effectively named species *Amycolatopsis flava* which groups with them in 16S rRNA trees (Wei et al., 2015). In agreement with a previous study where POCP was applied to a slightly smaller set of *Amycolatopsis* genomes (Adamek et al., 2018), less than 50% proteins were conserved between *A. halophila* YIM93223^T^ and the majority of the *Amycolatopsis* strains but with some exceptions (**Supplementary Table S2**). These results are consistent with the classification of *A. halophila* into a different genus (Nouioui et al., 2018). We also applied a stringent approach to identify conserved proteins by calculating the core genome for the entire dataset with a sequential exclusion of genomes from members of other genera and anomalous *Amycolatopsis* strains (**Figure 4**). However, no clear correlation to core conserved proteins and intergeneric boundary could be identified from this analysis.

The majority of the *Amycolatopsis* strains clustered into four robust groups based on the phylogenomic and taxogenomic analyses (**Figures 1, 2A–D**). The pan-genomic analyses identified some genes that are conserved within each group but absent in other *Amycolatopsis* strains (**Supplementary Tables S4A–D**). 147 genes were specific to Group A, 114 genes to Group B, 54 genes to Group C and 244 genes to group D. A large proportion of these genes (42–59%) encode hypothetical proteins; however, some genes are annotated as transcriptional regulators, Sigma factors and some genes potentially belong to different biosynthetic gene clusters. Indeed, the biosynthetic potential to produce secondary metabolites varied between the *Amycolatopsis* groups with a strong correlation to the number of biosynthetic gene clusters (Adamek et al., 2018).

## Conclusions

*Amycolatopsis* is a homogeneous genus where most strains conform to the phylogenomic and taxogenomic indices defined for intra-generic boundaries. In contrast, *A. palatopharyngis* DSM 44832^T^ and *A. marina* CGMCC4 3568^T^ formed a clade closer to *Saccharomonospora* strains with comparable taxogenomic distances between them and the other *Amycolatopsis* strains. We also show that genomic data are robust and reproducible between different laboratories and can help resolve cases of misclassification and misidentification. Some strains identified as *A. orientalis* should either be assigned to other species or to presumptive novel species. Genomic analyses also assigned some undefined strains to known species. These results provide further evidence that matrices derived from the whole genome sequencing data can provide a robust framework for prokaryotic systematics.

## Data Accession

The genome sequence data from this study has been submitted to GenBank/DDBJ/EMBL databases and are publically available with the accession numbers given in **Supplementary Table S1**.

## Author Contributions

VS, MG, IS, and H-PK have designed the study. VS and GT carried out the experimental work. VS and JB analyzed the data. VS drafted the manuscript. All the authors provided intellectual inputs and approved the final version.

## Conflict of Interest Statement

The authors declare that the research was conducted in the absence of any commercial or financial relationships that could be construed as a potential conflict of interest.

## Footnotes

1 http://www.bacterio.net/amycolatopsis.html
