# Supplementary material for: Revisiting the Taxonomic Status of the Biomedically and Industrially Important Genus Amycolatopsis, Using a Phylogenomic Approach
Source: Front Microbiol. 2018 Sep 27;9:2281. doi: 10.3389/fmicb.2018.02281 (PMC6171478; doi:10.3389/fmicb.2018.02281)
Supplement: Supplementary file 5 [file Data_Sheet_1.PDF]

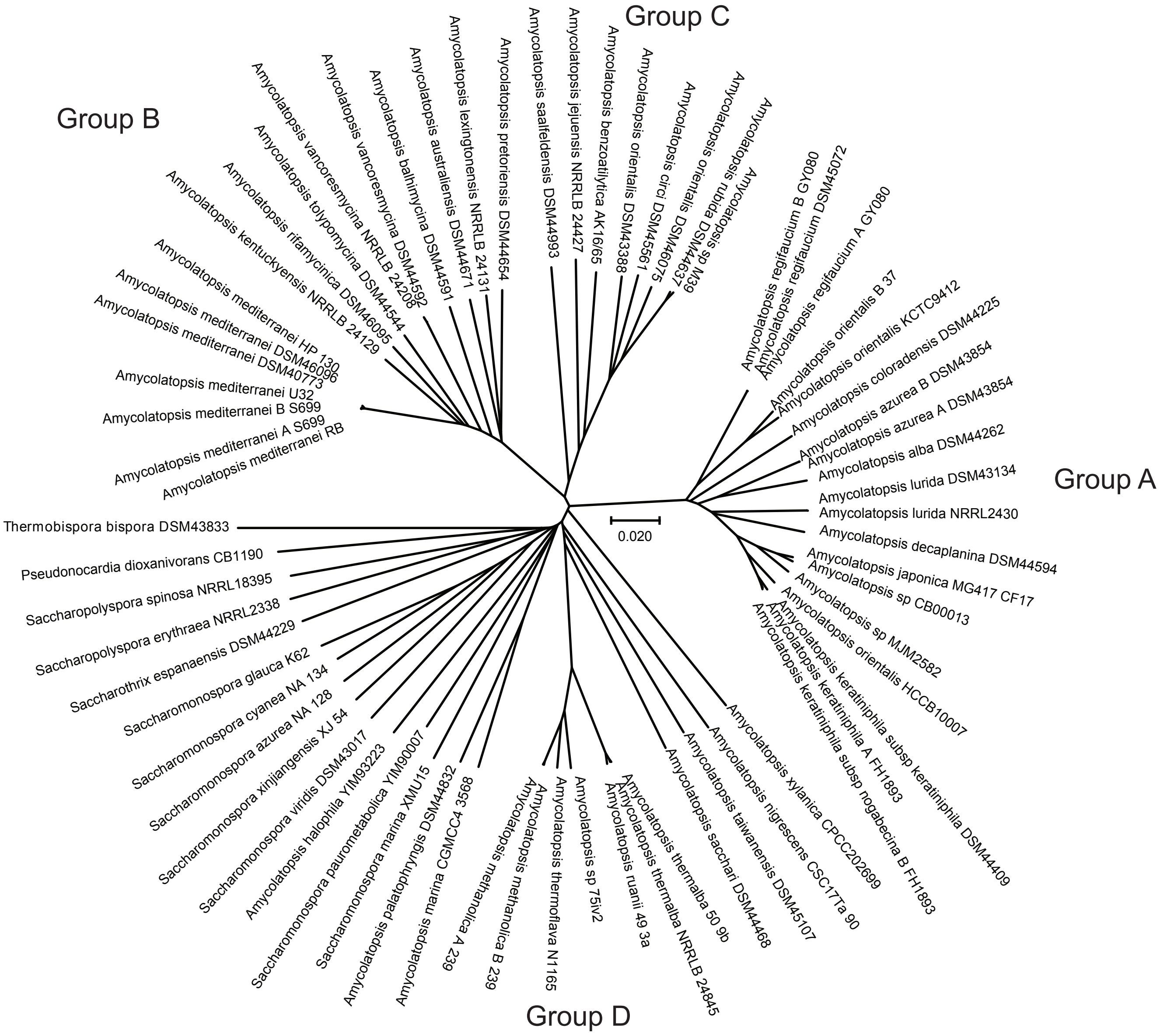

Supplementary Fig. 1. A Neighbor-Joining tree from the distance matrix obtained from pairwise GGDC analysis.
